# Direct transdifferentiation of spermatogonial stem cells to morphological, phenotypic and functional hepatocyte-like cells via the ERK1/2 and Smad2/3 signaling pathways and the inactivation of cyclin A, cyclin B and cyclin E

**DOI:** 10.1186/1478-811X-11-67

**Published:** 2013-09-18

**Authors:** Zhenzhen Zhang, Yuehua Gong, Ying Guo, Yanan Hai, Hao Yang, Shi Yang, Yang Liu, Meng Ma, Linhong Liu, Zheng Li, Wei-Qiang Gao, Zuping He

**Affiliations:** 1 Renji Hospital, Stem Cell Research Center, Shanghai Jiao Tong University School of Medicine, 1630 Dongfang Road, Shanghai 200127, China; 2 Department of Urology, Shanghai Human Sperm Bank, Renji Hospital, Shanghai Jiao Tong University School of Medicine, Shanghai 200001, China; 3 Shanghai Cancer Institute, State Key Laboratory of Oncogenes and Related Genes, Renji Hospital, Shanghai Jiao Tong University School of Medicine, Shanghai 200032, China; 4 Shanghai Key Laboratory of Reproductive Medicine, Shanghai 200025, China

**Keywords:** Spermatogonial stem cells, Direct transdifferentiation, Hepatic stem cells, Mature hepatocytes, Morphology and phenotype, Function, ERK1/2 and Smad2/3 signaling pathways

## Abstract

**Background:**

Severe shortage of liver donors and hepatocytes highlights urgent requirement of extra-liver and stem cell source of hepatocytes for treating liver-related diseases. Here we hypothesized that spermatogonial stem cells (SSCs) can directly transdifferentiate to hepatic stem-like cells capable of differentiating into mature hepatocyte-like cells *in vitro* without an intervening pluripotent state.

**Results:**

SSCs first changed into hepatic stem-like cells since they resembled hepatic oval cells in morphology and expressed *Ck8*, *Ck18*, *Ck7*, *Ck19*, OV6, and albumin. Importantly, they co-expressed CK8 and CK19 but not ES cell markers. Hepatic stem-like cells derived from SSCs could differentiate into small hepatocytes based upon their morphological features and expression of numerous hepatic cell markers but lacking of bile epithelial cell hallmarks. Small hepatocytes were further coaxed to differentiate into mature hepatocyte-like cells, as identified by their morphological traits and strong expression of *Ck8*, *Ck18*, *Cyp7a1*, *Hnf3b*, *Alb, Ta*t, *Ttr*, albumin, and CYP1A2 but not *Ck7* or CK19. Notably, these differentiated cells acquired functional attributes of hepatocyte-like cells because they secreted albumin, synthesized urea, and uptake and released indocyanine green. Moreover, phosphorylation of ERK1/2 and Smad2/3 rather than Akt was activated in hepatic stem cells and mature hepatocytes. Additionally, cyclin A, cyclin B and cyclin E transcripts and proteins but not cyclin D1 or *CDK1* and *CDK2* transcripts or proteins were reduced in mature hepatocyte-like cells or hepatic stem-like cells derived from SSCs compared to SSCs.

**Conclusions:**

SSCs can transdifferentiate to hepatic stem-like cells capable of differentiating into cells with morphological, phenotypic and functional characteristics of mature hepatocytes via the activation of ERK1/2 and Smad2/3 signaling pathways and the inactivation of cyclin A, cyclin B and cyclin E. This study thus provides an invaluable source of mature hepatocytes for treating liver-related diseases and drug toxicity screening and offers novel insights into mechanisms of liver development and cell reprogramming.

## Introduction

Liver cancer is one of most common tumors around the world and the majority of patients with this disease usually die within one year [1]. Hepatitis B virus infected over 300 million people, which is a common cause of end-stage liver diseases including cirrhosis [2]. The effective treatment for end-stage liver diseases is liver transplantation [3]. However, there is a severe shortage of liver donors, which is the major obstacle for treatment of patients with end-stage liver diseases. Consequently, many patients suffering from end-stage liver diseases have to be on the waiting list and they die before liver transplantation can be performed. Hepatocytes’ transplantation is an alternative approach to restore liver function and cure liver congenital metabolic diseases [4,5]. Nevertheless, human hepatocytes are scarce in number and have a very limited potential of proliferation. Therefore, it is crucial to seek a readily available source of hepatocytes from extra-liver tissues and/or stem cells that can be cultured and expanded *in vitro* to treat patients with end-stage liver diseases.

Hepatic stem cells can differentiate into functional hepatocytes [6]. Nevertheless, the number of hepatic stem cells is very few in patients with end-stage liver diseases. Embryonic stem (ES) cells have been used to differentiate into hepatocytes [7]. However, the availability of human ES cells is rather limited due to the ethic and safety issues [8]. Recently, the induced pluripotent stem (iPS) cells have been utilized to generate functional hepatocytes [9,10]. Nevertheless, it is cautious to use hepatocytes derived from iPS cells for clinical applications due to their genetic instability and using viral transduction for reprogramming somatic cells to pluripotency, which poses a potential tumor risk that could limit their use in regenerative medicine. Adult tissue stem cells can differentiate into mature cells with specific functions. One obvious advantage of using adult tissue stem cells is that there is no ethical issue compared to ES cells, and most importantly, certain adult tissue stem cells have multipotency to differentiate into various kinds of cells for regenerative medicine.

Spermatogonial stem cells (SSCs) are a subpopulation of type A spermatogonia in the testis. SSCs were previously regarded as unipotent stem cells since they were thought to differentiate into sperm only. However, this concept has recently been changed. Notably, recent studies have demonstrated that SSCs from both mouse and human testes can de-differentiate to become ES-like cells that can differentiate into various cell lineages of all three embryonic germ layers [11,12], suggesting that SSCs have important implications in regenerative medicine. On the other hand, SSCs de-differentiate to become pluripotent ES-like cells, which may cause tumor since ES-like cells can form teratomas after transplantation. Recent study suggests that SSCs transdifferentiate into prostatic, uterine, and skin epithelium *in vivo* after transplantation [13]. However, it remains unknown whether SSCs have the potential to transdifferentiate into other types of stem cells *in vitro*. In this study, we propose a novel concept that SSCs can directly transdifferentiate to hepatic stem cells *in vitro* capable of differentiating into mature hepatocytes, which achieves two significant endpoints. First of all, direct transdifferentiation of primary SSCs to hepatic stem cells without the process of de-differentiation to pluripotent ES-like cells and embryonic body formation could simplify the reprogramming procedure. Secondly, our direct programming of transdifferentiation using growth factors without gene transduction could be much safer to generate mature hepatocytes for cell therapy of chronic liver disease and metabolic abnormalities. Here we present detailed induction and differentiation protocols as well as molecular and cellular evidence supporting direct transdifferentiation of SSCs into hepatic stem-like cells that are able to differentiate into cells with morphological, phenotypic, and functional mature hepatocyte-like cells via the activation of ERK1/2 and Smad2/3 pathways.

## Materials and methods

### Spermatogonial stem cell line C18-4 cells and culture

Spermatogonial stem cell line, namely C18-4 cells, was established by transfecting mouse SSCs with a plasmid expressing the SV40 large T antigen [14]. C18-4 cells were cultured with Dulbecco’s Modified Eagle’s Medium/Nutrient Mixture F12 (DMEM/F12, Gibico, Grand Island, NY) supplemented with 10% fetal bovine serum (FBS, Gibico), 2 mM L-glutamine (Invitrogen, Carlsbad, CA), and 100 unit/ml penicillin and streptomycin (Invitrogen). The cells were passed every 3-4 days and maintained at 34°C in a humidified 5% CO_2_ incubator.

### Transdifferentiation of SSCs to hepatic stem-like cells and mature hepatocyte-like cells

Primary SSCs were isolated from the testes of 6-day-old BALB/c mice using two-step enzymatic digestion and magnetic activated cell sorting with an antibody to GFRA1 according to procedure as described previously [15]. All animal care procedures were performed pursuant to the National Research Council’s Guide for the Care and Use of Laboratory Animals, China. Experimental protocols used were approved by the Renji Hospital Animal Care and Use Committee.

**Figure 1:**
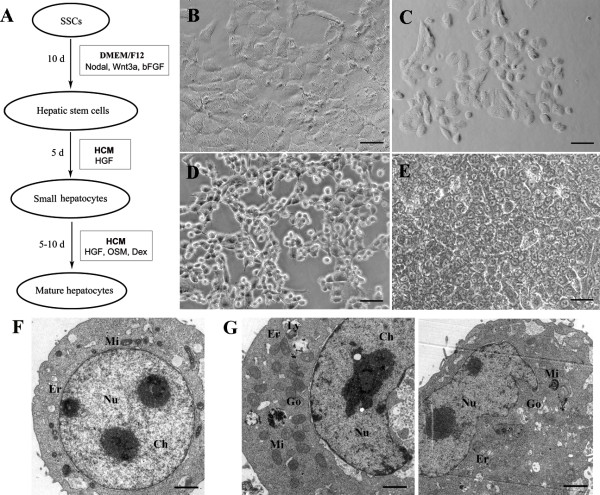
**A novel protocol for transdifferentiation of SSCs to mature hepatocyte-like cells and their morphological features. (A)** Schematic diagram showed the procedures for inducing SSCs to transdifferentiate to mature hepatocyte-like cells. **(B**-**E)** Morphology was characterized for SSCs **(B)**, hepatic stem-like cells **(C)**, small hepatocytes **(D)**, and mature hepatocyte-like cells **(E)**. Scale bars in **B**, **C**, **D**, and **E** = 50 μm. **(F**-**G)** Ultrastructure of hepatic stem cells **(F)** and mature hepatocyte-like cells **(G)** from SSCs. Note: mitochondria (Mi), endoplasmic reticulum (Er), lysosome (Ly), Golgi apparatus (Go), and chromatin (Ch), nucleus (Nu). Scale bars in **F** and **G**= 2 μm.

The C18-4 cells and primary SSCs were seeded at a density of 5,000 cells/well in 24-well plates with DMEM/F12 containing FBS and the combinations of 2 or 3 growth factors, including Activin A (Peprotech, Rocky Hills, NJ), Nodal (R&D System, Minneapolis, MN), Wnt3a (R&D System,), and bFGF (Peprotech). To optimize transdifferentiation of SSCs to hepatic stem-like cells, we used 6 culture conditions including the following components: i) 10% FBS; ii) 50 ng/ml Activin A + 50 ng/ml Wnt3a; 3) 50 ng/ml Nodal + 50 ng/ml Wnt3a; iv) 50 ng/ml Nodal + 50 ng/ml Wnt3a + 20 ng/ml bFGF + liver extract; v) 50 ng/ml Activin A + 50 ng/ml Wnt3a + liver extract; vi) 50 ng/ml Nodal + 50 ng/ml Wnt3a + liver extract. The best culture condition for transdifferentiation of SSCs to hepatic stem-like cells was as follows: DMEM/F12 supplemented with 0.5% FBS, 50 ng/ml Nodal, 50 ng/ml Wnt3a, and 20 ng/ml bFGF as illustrated in Figure 1A. Various FBS concentrations, including 0.5%, 2%, and 10%, were used to optimize the results. When C18-4 cells were seeded at a density of 10^4^cells/ml, 2% FBS were used for their proliferation for 2 or 3 days, and FBS concentration was reduced to 0.5% to restrain the overgrowth of C18-4 cells. For transdifferentiation assays, the medium was refreshed every 2 days and the cells were cultured for 10 days.

For further differentiation, transdifferentiated cells were cultured on 0.1% gelatin-coated tissue culture dishes with hepatocyte culture medium (HCM) plus EGF supplement (BD Bioscience, Bedford, MA) and 20 ng/ml hepatocyte growth factor (HGF, Peprotech). Medium was changed every 2 days and the cells were cultivated for 5 days. The differentiated cells were matured for another 5-10 days using HCM supplemented with 10 ng/ml HGF, 10 ng/ml Oncostain M (OSM, Peprotech), and 10^-4^ mM dexamethasone (Dex, Sigma-Aldrich, St. Louis, MO).

### Transmission electron microscopy (TEM)

Hepatic stem-like cells and mature hepatocyte-like cells derived from SSCs were fixed in 2.5% w/v glutaraldehyde in 0.1 M cacodylate buffer. After extensive washing in PBS, the cells were post-fixed in 1% w/v OsO4 for 30 min, dehydrated in a graded solution of ethanol and embedded in Epon. Ultrathin sections were cut and examined under an electron microscope after staining with uranyl acetate and lead citrate.

### Immunocytochemistry

Immunocytochemistry was performed on SSCs, the transdifferentiated cells, and the fully differentiated cells according to the procedure described previously [16]. The cells were fixed with 4% paraformaldehyde and permeabilized in 0.4% triton-X 100 (Sigma-Aldrich) for 15 min. After washing with phosphate-buffered saline (PBS, Gibico), cells were blocked in 1% bovine serum albumin (BSA, Sigma-Aldrich) for 15 min and followed by incubation with primary antibodies at a dilution with 1:100 overnight at 4°C. Primary antibodies used were anti-VASA (Santa Cruz Biotechnology Inc., Santa Cruz, CA), anti-RET (Santa Cruz), anti-UCHL1 (BD Bioscience), anti-GFRA1 (Santa Cruz), anti-OV6 (R&D System), anti-CYP1A2 (Santa Cruz), anti-ALB (Novus Biologicals, Littleton, CO), anti-PLZF (abcam, Cambridge, MA), anti-SSEA-1(Chemicon, Temecula, CA), anti-SSEA-4 (Chemicon), anti-Nanog (Chemicon), or anti-TRA-1-81(Chemicon). After three washes with PBS, the cells were incubated with the secondary antibody, including FITC-conjugated or rhodamine-conjugated IgG (Jackson ImmunoResearch, West Grove, PA), at a 1:200 dilution for 45 min at room temperature. DAPI (4′-6-diamidino-2-phenylindole) was used to stain the nuclei, and the cells were observed for epifluorescence using fluorescence microscope (Nikon Eclipse Ti-S, Nikon Corporation, Tokyo, Japan). Double staining was performed to determine whether the cells derived from SSCs were co-expressing CK19 and CK8 using anti-CK19 (R&D System) and anti-CK8 (Santa Cruz).

Immunofluorescence was also carried out to determine the expression changes of phosphorylation of ERK1/2, Smad2, Stat3, and Akt in the C18-4 cells, the transdifferentiated cells, and differentiated cells using antibodies against phospho-ERK1/2 (Cell Signaling Technology, Inc., Danvers, MA), phospho-Smad2 (Cell Signaling Technology), phospho-Stat3 (Cell Signaling Technology), or phospho-Akt (Cell Signaling Technology).

### RNA extraction and reverse transcription-polymerase chain reaction (RT-PCR)

Total RNA was extracted from C18-4 cells, the transdifferentiated cells, and the fully differentiated cells from C18-4 cells and primary SSCs using Trizol (Invitrogen). Reverse transcription (RT) was performed using First Strand cDNA Synthesis Kit (Fermentas, Lithuania) and PCR was performed according to the protocol as described previously [17]. The forward and reverse primers and PCR products of the chosen genes, including *Cytokine 8 (Ck8)*, *Ck18*, *Ck7*, *Ck19*, *Cyp1a2*, *Cyp7a1*, *hepatocyte nuclear factor* (*Hnf*)*3b*, *Hnf4a*, *Albumin* (*Alb*), *tyrosine aminotransferase (Tat)*, transthyretin (*Ttr*), *Cyclin A*, *Cyclin B, Cyclin D1, Cyclin E, CDK1, CDK2, c-fos, Oct-4*, and *Gapdh* were designed and listed in Additional file 1: Table S1. The PCR reaction started at 94°C for 5 min and was performed as follows: denaturation at 94°C for 30 sec, annealing at a temperature (Tm) as indicated in Additional file 1: Table S1 for 45 sec, and elongation at 72°C for 45 sec. After 30 cycles, the samples were incubated for an additional 5 min at 72°C. PCR products were separated by electrophoresis on 1.2% agarose gels. The gels were exposed to chemiluminescence (Chemi-Doc XRS, Bio-Rad, Hercules, CA).

### Flow cytometry

Hepatic stem-like cells derived from SSCs in the conditioned medium with or without MEK1 inhibitor PD98059 were first fixed with 1% paraformaldehyde, permeabilized by permeabilization buffer (eBioscience, San Diego, CA), and incubated with primary antibody to CK8. After washes, cells were incubated by FITC-coupled secondary antibody, and analyses were performed using Accuri C6 flow cytometer (Accuri Cytometers, Ann Arbor, MI) and Cflow software (Accuri Cytometers). The SSCs-derived cells without primary antibody but were incubated by FITC-coupled secondary antibody served as a negative control.

### Western blots

Cells were lysed with RIPA buffer (Santa Cruz) for 30 min on ice. After 30 min lysis on ice, cell lysates were cleared by centrifugation at 12,000 g, and the concentration of protein was measured by BCA kit (Dingguo Company, China). Ten micrograms of cell lysate from each sample were used for SDS-PAGE (Bio-Rad Laboratories, Richmond, CA), and Western blots were performed according to the protocol we described previously [16]. The chosen antibody included CK8, phos-ERK1/2 (Santa Cruz), phos-Smad2 (Santa Cruz), Smad2/3 (Santa Cruz), cyclin A (Santa Cruz), cyclin B (Santa Cruz), cyclin D1 (Santa Cruz), cyclin E (Santa Cruz), and ACTB (beta-actin) (IMGENEX Corp). After extensive washes in PBS, the blots were detected by chemiluminescence (Chemi-Doc XRS, Bio-Rad, Hercules, CA).

### Albumin synthesis of hepatocyte-like cells derived from SSCs by ELISA

Primary mouse hepatocytes were isolated from liver tissues using 0.03% collagenase IV and 0.025 EDTA and cultured in William’s E + 100 nM insulin + 15% FBS according to procedure as described previously [18]. Culture medium from SSCs and the differentiated cells was collected over 2 days from equivalent numbers of cells. Albumin production in the medium from the differentiated cells and primary mouse hepatocytes was determined by mouse Albumin ELISA Quantitation Kit (Alpha Diagnostic Intl. Inc, San Antonio, TX) according to the manufacturer’s instructions. Albumin secretion was normalized to per 10^5^ cells.

### Urea assays of hepatocyte-like cells derived from SSCs

After exposure of the cells to 2 mM ammonium chloride (Sigma-Aldrich) for 24 h, urea productions in the culture media of SSCs, mature hepatocyte-like cells derived from SSCs, and primary mouse hepatocytes were measured using Urea Assay Kit (Biovision, Mountain View, CA). Fresh culture medium supplemented with 2 mM ammonium chloride was used as a negative control. Urea production was expressed as mM urea nitrogen per 10^5^ cells within 24 h.

### Uptake and release of indocyanine green (ICG) of hepatocyte-like cells derived from SSCs

Indocyanine green (ICG) (Sigma-Aldrich) was suspended in DMSO (Sigma-Aldrich) for a stock at 100 mg/ml and freshly diluted in HCM to a working concentration of 1 mg/ml. Hepatocyte-like cells derived from SSCs and primary mouse hepatocytes were incubated with the diluted ICG for 30 min at 37°C. After extensive washes, positive foci were counted and photographed under the microscope, and the cells were returned to HCM and incubated for 20 h. Release of cellular ICG stain was examined, and undifferentiated C18-4 cells were used as a negative control while primary mouse hepatocytes served as a positive control.

### Statistical analysis

All experiments were performed independently at least 3 times. All the values were presented as mean ± SEM, and statistically significant differences (p< 0.05) between SSCs and differentiated cells were determined using the analysis of variance (ANOVA) and a 2-tailed *t*-test.

## Results

### Direct transdifferentiation of SSCs to hepatic stem-like cells

We first verified the identity of the C18-4 cells using various markers for germ cells and SSCs. Immunocytochemistry revealed that C18-4 cells expressed VASA (Additional file 2: Figure S1A), UCHL1 (Additional file 2: Figure S1B), GFRA1 (Additional file 2: Figure S1C), and RET (Additional file 2: Figure S1D), suggesting that the C18-4 cells are phenotypically SSCs.

To induce the transdifferentiation of C18-4 cells to hepatic stem-like cells, 6 conditioned media supplemented with various growth factors and liver tissue extract (Figure 1A and Additional file 3: Figure S2B-2F) were employed. Among these culture conditions, the combination of growth factors, including Nodal, Wnt3a, and bFGF, was the best approach for inducing the transdifferentiation of C18-4 cells. Other approaches led to a high death rate of the cells (Additional file 3: Figure S2C, 2D, and 2F), or had little effect on transdifferentiation (Additional file 3: Figure S2B and 2E). The C18-4 cells proliferated rapidly when cultured with 10% FBS (Additional file 3: Figure S2A). To avoid cell overgrowth, culture medium was changed into a low concentration of FBS (0.5-2%) in DMEM/F12 supplemented with Nodal, Wnt3a, and bFGF (Figure 1B). After 10 days of culture, the morphology of cells was obviously changed and became oval and stereoscopic in shape (Figure 1C), which is similar to hepatic oval cells (termed hepatic stem cells). Transmission electron microscopy revealed that these cells had an ovoid nucleus with condensed chromatin, a higher ratio of nuclei to cytoplasm, and few organelles including immature mitochondria (Mi) and rough endoplasmic reticulum (Er) (Figure 1F). Compared to mature hepatocytes, oval cells were relatively small since they had a median diameter of 8 μm.

**Figure 2:**
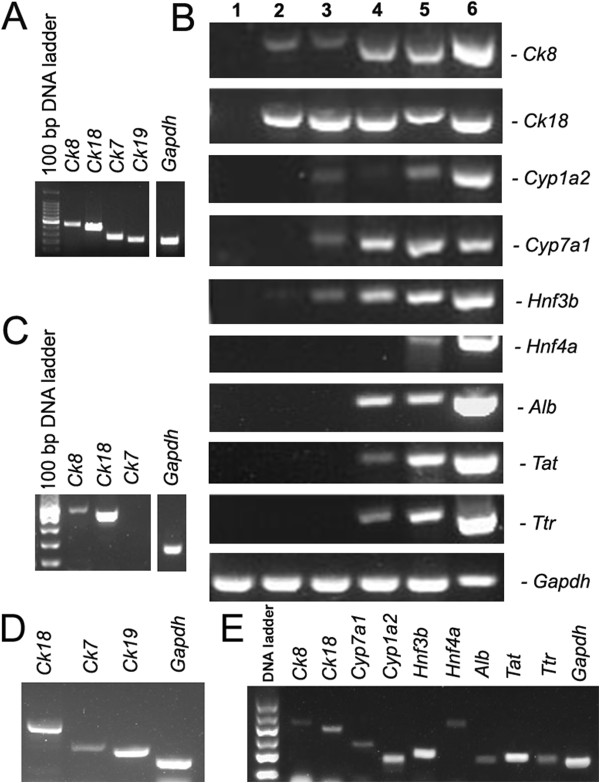
**Transcriptional characteristics of transdifferentiation of SSCs into hepatic stem-like cells and hepatocyte-like cells. (A)** RT-PCR revealed mRNA expression of *Ck8*, *Ck18*, *Ck7*, and *Ck19* in hepatic stem-like cells derived from SSCs. **(B)** RT-PCR showed the transcripts of *Ck8*, *Ck18*, *Cyp1a2*, *Cyp7a1*, *Hnf3b*, *Hnf4a*, *Alb*, *Ta*t, and *Ttr* in SSCs (lane 1), SSC induction for 7 days (lane 2), SSC induction for 10 days (lane 3), small hepatocytes (lane 4), and mature hepatocyte-like cells (lane 5) derived from SSCs. The expression of these genes in liver tissues of adult mice (lane 6) was used as positive controls. **(C)** The transcription of *Ck8*, *Ck18*, and *Ck7* in mature hepatocyte-like cells derived from SSCs. *Gapdh* was used as loading controls of total RNA. **(D)** The transcription of *Ck18*, *Ck7* and *Ck19* in the cells derived from primary mouse SSCs. *Gapdh* was used as loading controls of total RNA. **(E)** RT-PCR showed the transcripts of *Ck8*, *Ck18*, *Cyp1a2*, *Cyp7a1*, *Hnf3b*, *Hnf4a*, *Alb*, *Ta*t, and *Ttr* in mature hepatocyte-like cells derived from primary SSCs. *Gapdh* was used as loading controls of total RNA.

**Figure 3:**
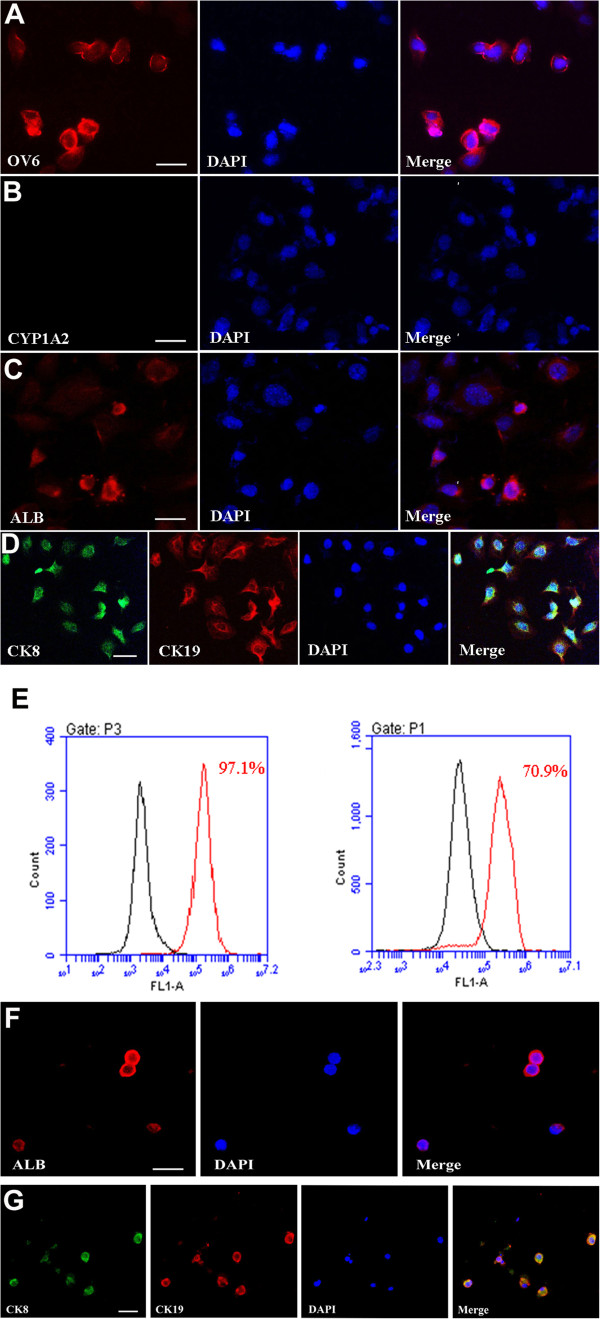
**The expression of OV6, CYP1A2, ALB, CK8, and CK19 of hepatic stem-like cells derived from transdifferentiation of SSCs. (A**-**D)** Immunocytochemistry showed protein expression of OV6 **(A)**, CYP1A2 **(B)**, ALB **(C)**, as well as co-expression of CK8 and CK19 **(D)** in transdifferentiated cells derived from C18-4 cells. Scale bars in **A**, **B**, **C**, and **D** = 20 μm. **(E)** Flow cytometry showed expression of CK8 in the transdifferentiated cells derived from C18-4 cells in the conditioned medium without PD98059 (left panel) or with PD98059 (right panel). **(F**-**G)** Immunocytochemistry showed protein expression of ALB **(F)** as well as co-expression of CK8 and CK19 **(G)** in transdifferentiated cells derived from mouse primary SSCs. Scale bars in **F** and **G** = 20 μm.

We next analyzed phenotypic characteristics of the cells derived from SSCs at transcriptional and translational levels in order to clarify their identity. We examined the gene expressions of hepatocytic and cholangiocytic markers by RT-PCR analysis. As shown in Figure 2A, the cells derived from C18-4 cells expressed the transcripts of *Ck8* and *Ck18*, markers for hepatic cells [19], as well as *Ck7* and *Ck19*, hallmarks for bile epithelial cells [19], thereby demonstrating their bipotency. Notably, the cells derived from primary SSCs expressed the transcripts of *Ck18*, *Ck7* and *Ck19* (Figure 2D), further verifying that SSCs could be converted to bipotential hepatic stem cells. The expression of *Hnf4a*, *Alb*, *Tat*, and *Ttr* was undetected, while *Cyp1a2*, *Cyp7a1*, and *Hnf3b* transcripts were weakly found in these cells after 7-10 days of transdifferentiation (Figure 2B, lanes 2 and 3). Immunocytochemistry further showed that the cells obtained from C18-4 cells were strongly positive for OV6, a specific marker for hepatic stem cells [19,20], (Figure 3A) and ALB (Figure 3C) but not CYP1A2 (Figure 3B). In contrast, the expression of germ cell and SSCs markers, including VASA (Additional file 4: Figure S3A), RET (Additional file 4: Figure S3B), and PLZF (Additional file 4: Figure S3D), was undetectable in the cells derived from SSCs, while GFRA1 expression was remarkably reduced in these cells (Additional file 4: Figure S3C) compared to SSCs (Additional file 2: Figure S1C). Significantly, the expression of ES cell markers including SSEA-1 (Additional file 5: Figure S4A), SSEA-4 (Additional file 5: Figure S4B), Nanog (Additional file 5: Figure S4C), and TRA-1-80 (Additional file 5: Figure S4D), was not detected in these cells derived from SSCs, implicating that these cells didn’t convert to ES cells. Double staining, using antibodies against CK8 and CK19, revealed that the cells generated from SSCs were co-expressing CK8 and CK19 (Figure 3D). Furthermore, the cells generated from primary mouse SSCs expressed ALB (Figure 3F) and were co-expressing CK8 and CK19 (Figure 3G). Collectively, these results suggest that SSCs could transdifferentiate into hepatic stem-like cells phenotypically. Notably, flow cytometry showed that over 97% of the cells derived from SSCs were positive for CK8 (Figure 3E, left panel), reflecting a rather high transdifferentiation efficiency of SSCs to hepatic stem-like cells. In contrast, only 70.9% of cells derived from SSCs were positive for CK8 when SSCs were pretreated with ERK1/2 upstream inhibitor PD98059 (Figure 3E, right panel), indicating that ERK1/2 signaling is involved in the transdifferentiation of SSCs to hepatic stem-like cells.

### Differentiation of SSCs-derived hepatic stem-like cells into small hepatocytes

**Figure 4:**
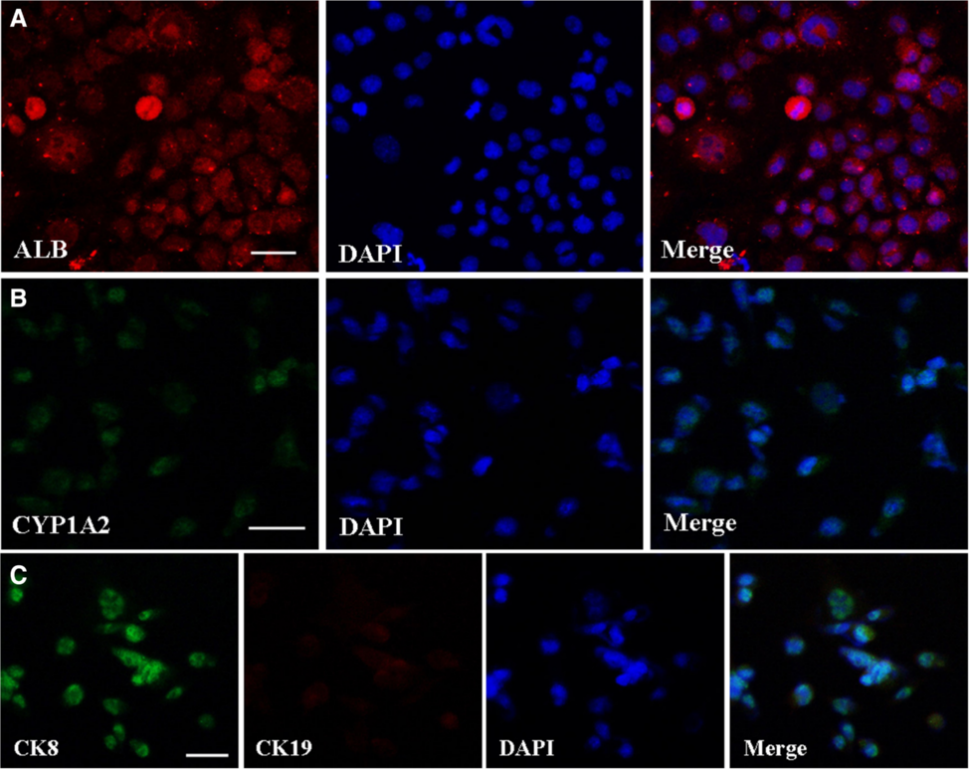
**Translational characterization of small hepatocytes derived from SSCs. (A**-**C)** Immunocytochemistry displayed expression of ALB **(A)**, CYP1A2 **(B)**, as well as co-expression of CK8 and CK19 **(C)** in small hepatocytes derived from SSCs. Scale bars in **A**, **B**, and **C** = 20 μm.

After 10 days of transdifferentiation, the number of cells became double or triple compared to the cells seeded. We next induced the differentiation of hepatic stem-like cells derived from SSCs using HCM and HGF. Morphological characteristic is an important means of distinguishing small hepatocytes from hepatic stem-like cells. Under phase-contrast microscopy, hepatic stem-like cells derived from SSCs was changed to become round in shape with a high ratio of nucleus to cytoplasm (Figure 1D), which was similar to small hepatocytes. We also examined phenotypic characteristics of the cells derived from hepatic stem-like cells in order to probe their identity. As shown in Figure 2B, lane 4, the cells derived from hepatic stem-like cells expressed *Ck8*, *Ck18*, *Cyp7a1*, *Hnf3b*, and *Alb* transcripts at high levels*,* whereas *Ck19* transcripts were significantly reduced in these cells (Additional file 6: Figure S5A, lane 4)*,* implicating that these cells possess the characteristics of small hepatocytes in phenotypes. Immunocytochemistry further revealed that the cells derived from hepatic stem-like cells strongly expressed ALB (Figure 4A) and had weak expression of CYP1A2 (Figure 4B). Double staining using antibodies against CK8 and CK19 revealed that the cells derived from hepatic stem-like cells expressed CK8 but not CK19 (Figure 4C). Together, our data implicate that hepatic stem-like cells derived from SSCs differentiate into small hepatocytes morphologically and phenotypically.

### Differentiation of SSCs-derived hepatic stem-like cells into mature hepatocyte-like cells

Hepatic stem cells are defined in part by their ability to differentiate into mature hepatocytes. We made endeavors to further induce the differentiation of hepatic stem-like cells derived from SSCs using HCM supplemented with HGF, OSM, and Dex. In morphology, small hepatocytes derived from SSCs changed to become polygonal appearance with tight cell-cell contacts and a low ratio of cellular nucleus to cytoplasm (Figure 1E), which showed an attribute of mature hepatocytes. In addition, our ultrastructural observations provided more convincing evidence that small hepatocytes further differentiated into mature hepatocyte-like cells since they contained well-developed organelles such as mitochondria (Mi), endoplasmic reticulum (Er), lysosome (Ly) and Golgi apparatus (Go) (Figure 1G).

**Figure 5:**
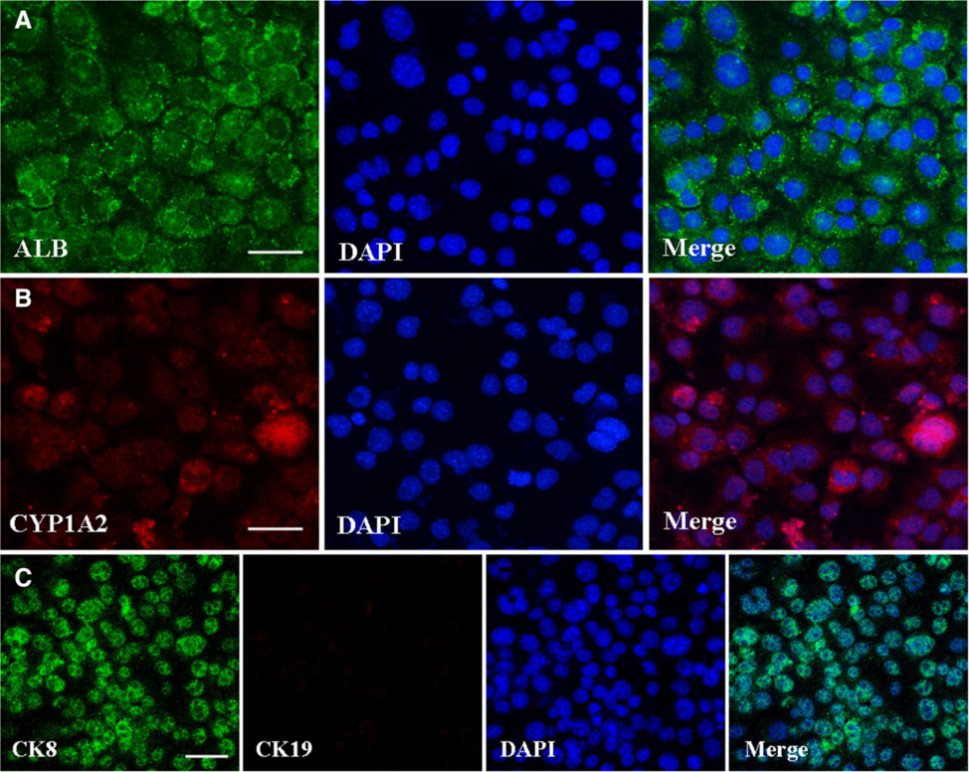
**Characterization of mature hepatocyte-like cells derived from SSCs. (A**-**C)** Immunocytochemistry showed expression of ALB **(A)**, CYP1A2 **(B)**, as well as co-expression of CK8 and CK19 **(C)** in mature hepatocyte-like cells derived from SSCs. Scale bars in **A**, **B**, and **C** = 50 μm.

We probed phenotypic characteristics of the fully differentiated cells derived from C18-4 cells and primary SSCs at transcriptional and translational levels. As shown in Figure 2B, lane 5, and Figure 2E, the cells derived from C18-4 cells and primary SSCs had strong mRNA expression of *Ck8*, *Ck18*, *Cyp7a1*, *Hnf4a*, *Hnf3b*, *Alb, Ta*t, and *Ttr*, whereas *Ck7* transcript was undetected in these cells (Figure 2C), reflecting that they possess the characteristics of mature hepatocytes in phenotypes. Immunocytochemistry further revealed that the fully differentiated cells derived from SSCs expressed ALB (Figure 5A) and CYP1A2 (Figure 5B) at high levels. Double staining, using antibodies against CK8 and CK19, showed that these cells derived from SSCs had strong expression of CK8 but not CK19 (Figure 5C). Considered together, our results implicate that the fully differentiated cells derived from SSCs differentiate into mature hepatocyte-like cells with morphologic and phenotypic features.

### Functional assays of mature hepatocyte-like cells derived from SSCs

**Figure 6:**
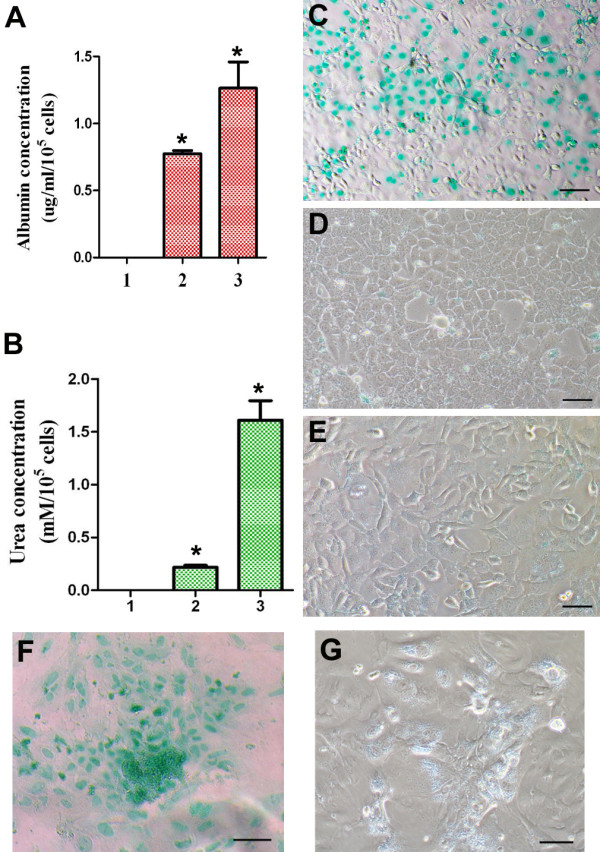
**Albumin synthesis, urea productions, and cellular uptake and release of ICG of mature hepatocyte-like cells derived from SSCs. (A)** ELISA showed albumin synthesis of SSCs (lane 1), mature hepatocyte-like cells derived from SSCs (lane 2), and primary hepatocytes (lane 3). “*” indicated statistically significant differences (p< 0.05) between SSCs and mature hepatocyte-like cells derived from SSCs or primary hepatocytes. **(B)** Urea assay displayed urea production of SSCs (lane 1), mature hepatocyte-like cells derived from SSCs (lane 2), and primary hepatocytes (lane 3). “*” indicated statistically significant differences (p< 0.05) between SSCs and mature hepatocyte-like cells derived from SSCs or primary hepatocytes. **(C**-**G)** Cellular uptake **(C)** and release **(D)** of ICG in mature hepatocyte-like cells derived from SSCs, uptake of ICG in SSCs **(E)**, as well as uptake **(F)** and release **(G)** of ICG in mouse primary hepatocytes. Scale bars in **C**-**G** = 50 μm.

We measured albumin synthesis, urea production, and indocyanine green (ICG) uptake and release to determine whether SSCs-derived cells with the morphological and phenotypic characteristics of hepatocytes also had functional attributes of hepatocytes. Albumin production is a specific test for the presence and metabolic activity of hepatocytes. ELISA showed that the differentiated cells from SSCs synthesized albumin (0.773 ± 0.0247 mg/ml/10^5^cells) (Figure 6A), which was comparable to albumin production of mouse primary hepatocytes (Figure 6A). Urea production is another characteristic of hepatocytes’ activity. Urea assay revealed that the differentiated cells from SSCs produced urea (0.217 ± 0.0188 mM/10^5^cells) (Figure 6B), which was lower than urea production of mouse primary hepatocytes (Figure 6B). Excretion of endogenous and exogenous compounds from the circulation is a major function of liver, and thus we examined this function in the SSCs-derived hepatocyte-like cells using ICG assay. As shown in Figure 6, ICG-positive signal was clearly seen in the cells derived from SSCs (Figure 6C), whereas ICG uptake was not detectable in the SSCs without treatment (Figure 6E). Cell counts indicated that about 40% SSCs-derived cells were positive for ICG. Furthermore, the ICG uptake in the SSCs-derived hepatocyte-like cells was excreted by 20 h after removal of ICG from culture medium (Figure 6D). Interestingly, the uptake and release of ICG from SSCs-derived hepatocyte-like cells (Figure 6C and D) was similar to those of mouse primary hepatocytes (Figure 6F-G).

### ERK1/2 and Smad2/3 but not Akt signaling pathways were activated during the transdifferentiation of SSCs into mature hepatocyte-like cells

**Figure 7:**
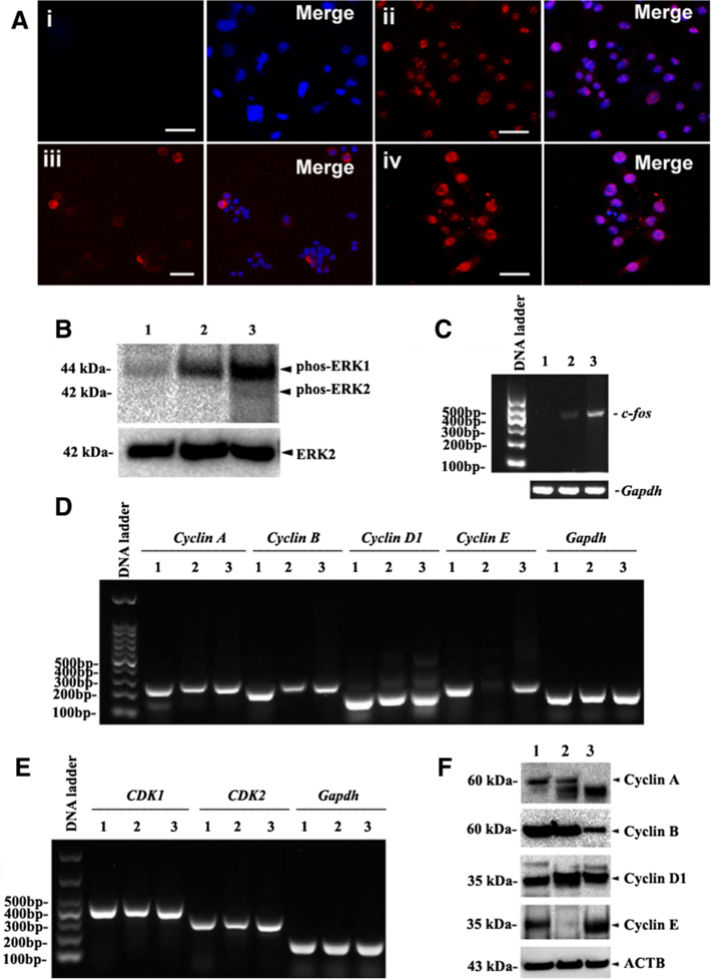
**Expression of ERK1/2 phosphorylation, *c-fos *transcription and cell cycle proteins in SSCs as well as in hepatic stem-like cells and mature hepatocyte-like cells derived from SSCs. (A)** Immunocytochemistry revealed the expression of phosph-ERK1/2 in SSCs (Panel i), hepatic stem-like cells derived from SSCs (Panel ii), small hepatocytes derived from SSCs (Panel iii), and mature hepatocyte-like cells derived from SSCs (Panel iv). Scale bars in **A** = 50 μm. **(B)** Western blots showed the expression of ERK1/2 phosphorylation in SSCs (lane 1), hepatic stem-like cells derived from SSCs (lane 2), and mature hepatocyte-like cells derived from SSCs (lane 3). The expression of ERK2 was used as a loading control of total proteins. **(C)** RT-PCR revealed the transcription of *c-fos* mRNA in SSCs (lane 1), hepatic stem-like cells derived from SSCs (lane 2), and mature hepatocyte-like cells derived from SSCs (lane 3). Housekeeping gene *Gapdh* served as a loading control of total RNA. **(D)** RT-PCR displayed mRNA expression of *cyclin A*, *cyclin B*, *cyclin D1*, and *cyclin E* in SSCs (lane 1), hepatic stem-like cells derived from SSCs (lane 2), and mature hepatocyte-like cells derived from SSCs (lane 3). *Gapdh* was used as loading control of total RNA. **(E)** RT-PCR showed transcripts of *CDK1* and *CDK2* in SSCs (lane 1), hepatic stem-like cells derived from SSCs (lane 2), and mature hepatocyte-like cells derived from SSCs (lane 3). *Gapdh* served as loading control of total RNA. **(F)** Western blots revealed the expression of cyclin **A**, cyclin **B**, cyclin **D**1, and cyclin **E** in SSCs (lane 1), hepatic stem-like cells derived from SSCs (lane 2), and mature hepatocyte-like cells derived from SSCs (lane 3). The expression of ACTB was used as a loading control of total proteins.

To gain novel insights into the molecular mechanisms underlying the transdifferentiation of SSCs into mature hepatocyte-like cells, we analyzed what signaling pathways were activated. Compared to the control (Figure 7A, panel i), ERK1/2 phosphorylation was enhanced remarkably in hepatic stem-like cells (Figure 7A, panel ii), small hepatocytes (Figure 7A, panel iii), and mature hepatocyte-like cells derived from SSCs (Figure 7A, panel iv). Western blots further revealed that ERK1/2 phosphorylation was increased in hepatic stem-like cells and mature hepatocyte-like cells derived from SSCs compared with SSCs (Figure 7B).

**Figure 8:**
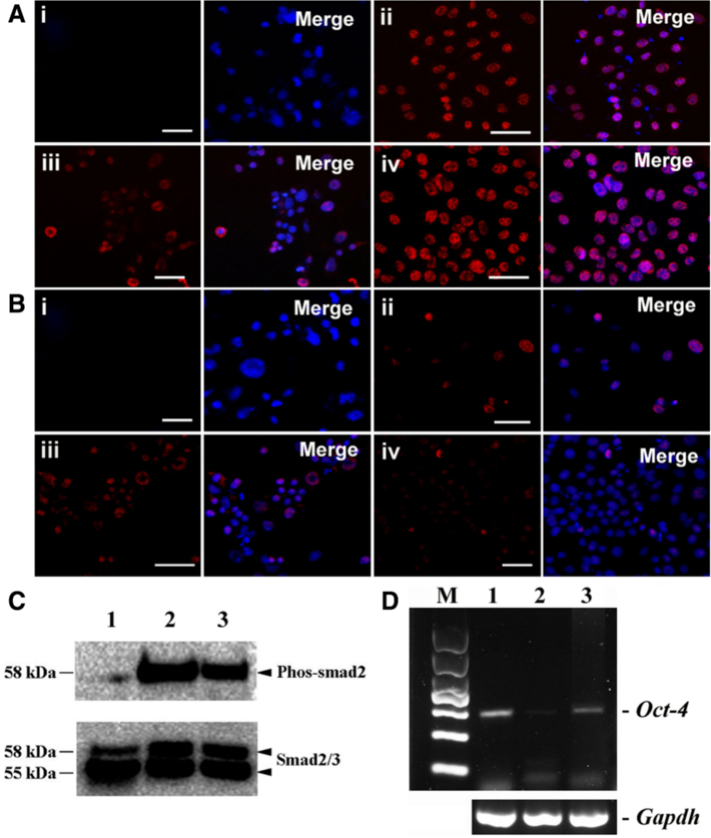
**Expression of Smad2-, and Stat3- phosphorylation and *Oct-4 *in SSCs, hepatic stem-like cells, small hepatocytes, and mature hepatocyte-like cells. (A)** Immunocytochemistry revealed expression of Expression of phosph-Smad2 in SSCs (Panel i), hepatic stem-like cells (Panel ii), small hepatocytes (Panel iii), and mature hepatocyte-like cells (Panel iv). **(B)** Expression of phosph-Stat3 in SSCs (Panel i), hepatic stem-like cells (Panel ii), small hepatocytes (Panel iii), and mature hepatocyte-like cells (Panel iv). Scale bars in **A**-**B** = 50 μm. **(C)** Western blots showed the expression of phosph-Smad2 in SSCs (lane 1), hepatic stem-like cells (lane 2), and mature hepatocyte-like cells (lane 3). **(D)** RT-PCR revealed *Oct-4* expression in SSCs (lane 1), hepatic stem-like cells (lane 2), and mature hepatocyte-like cells (lane 3).

In comparison to the control (Figure 8A, panel i), phosphorylation of Smad2 was elevated in hepatic stem-like cells (Figure 8A, panel ii), small hepatocytes (Figure 8A, panel iii) and mature hepatocyte-like cells (Figure 8A, panel iv) derived from SSCs. Additionally, phosphorylation of Stat3 was slightly increased in hepatic stem-like cells (Figure 8B, panel i) and small hepatocytes (Figure 8B, panel ii) but not in mature hepatocyte-like cells (Figure 8B, panel iv) derived from SSCs. Western blots further revealed that phosphorylation of Smad2 was enhanced in hepatic stem-like cells and mature hepatocyte-like cells compared with SSCs (Figure 8C). In direct contrast, phosphorylation of Akt was not detected in hepatic stem-like cells, small hepatocytes, or mature hepatocyte-like cells derived from SSCs (data not shown). These data suggest that ERK1/2 and Smad2 but not Akt signaling pathways contribute instructively to the transdifferentiation of SSCs into mature hepatocyte-like cells.

### *C-fos* and *Oct-4* transcripts were activated during the transdifferentiation of SSCs into mature hepatocyte-like cells

We also probed the *c-fos* transcript when SSCs transdifferentiated into mature hepatocyte-like cells. RT-PCR analysis showed that *c-fos* mRNA was increased significantly in mature hepatocyte-like cells derived from SSCs compared to hepatic stem-like cells or SSCs (Figure 7C), suggesting that *c-fos* transcript is activated during the transdifferentiation of mouse SSCs into mature hepatocyte-like cells. *Oct-4* transcript was reduced in hepatic stem-like cells compared to SSCs (Figure 8D), whereas it was enhanced in mature hepatocyte-like cells derived from SSCs compared to hepatic stem-like cells (Figure 8D).

### Cyclin A, cyclin B, and cyclin E but not cyclin D1, *CDK1*, or *CDK2* were inactivated during the transdifferentiation of SSCs into mature hepatocyte-like cells

We further explored the *cyclin A*, *cyclin B*, *cyclin D1*, *cyclin E, CDK1* and *CDK2* transcripts, as well as cyclin A, cyclin B, cyclin D1 and cyclin E proteins when SSCs transdifferentiated into mature hepatocyte-like cells. RT-PCR analysis revealed that mRNA of *cyclin A*, *cyclin B* and *cyclin E* was reduced significantly in mature hepatocyte-like cells derived from SSCs compared to hepatic stem-like cells or SSCs (Figure 7D), whereas there is no significant change in the transcripts of *cyclin D1*, *CDK1*, and *CDK2* (Figure 7D and E). Western blots further displayed that cyclin A, cyclin B and cyclin E proteins but not cyclin D1 protein were diminished in mature hepatocyte-like cells or hepatic stem-like cells derived from SSCs compared to SSCs (Figure 7E). Considered together, these data implicate that cyclin A, cyclin B, and cyclin E but not cyclin D1, *CDK1*, or *CDK2* are inactivated when mouse SSCs transdifferentiate into mature hepatocyte-like cells.

## Discussion

Lack of mature hepatocytes limits their wider application of hepatocyte transplantation and tissue engineering for the treatment of liver diseases. It is imperative to generate mature and functional hepatocytes independent of donor liver organs and from stem cells. Several studies have shown that hepatocytes can be generated from ES cells [7] and hepatic stem cells [6]. Recently, human iPS cells have been used to differentiate into hepatocytes [9,10]. However, hepatocytes derived from human iPS cells involve a complicated process. Of great concern, the use of iPS cells for cell therapies is hampered by their tumor-forming risk, due to reprogramming of somatic cells by gene transfer using viral vectors and their genetic instability. Therefore, more attention has been paid to generate hepatocytes from extra-liver source and adult stem cells. Here we have for the first time demonstrated that SSCs can directly transdifferentiate *in vitro* into the cells with morphological, phenotypic and functional attributes of mature hepatocyte-like cells.

We have previously identified the C18-4 cells as SSCs [16,17]. Here we further verified the identity of SSCs using a variety of markers for germ cells and SSCs. VASA has been recognized as a germ cell marker [21] while UCHL1 is a hallmark for spermatogonia [22]. GFRA1 and Ret are co-receptors for GDNF and markers for SSCs [23,24]. We found that C18-4 cells expressed VASA, UCHL1, GFRA1, and Ret. These data together with our previous studies indicate that C18-4 cells possess phenotypic characteristics of SSCs.

We used 6 different conditioned media to induce the transdifferentiation of SSCs. Among them, we optimized the defined condition with Nodal, Wnt3a, and bFGF for inducing the transdifferentiation of C18-4 cells into cells with morphological and phenotypic characteristics of hepatic stem-like cells. In morphology and ultrastructure, these cells derived from C18-4 cells resembled hepatic oval cells. In phenotypes, the cells derived from SSC line and primary SSCs expressed *Ck8*, *Ck18*, *Ck7*, and *Ck19*, and were co-expressing CK8 and CK19. CK8 and Ck18 are markers for hepatic cells while Ck7 and CK19 have been regarded as hallmarks for bile epithelial cells [19]. Moreover, the cells obtained from SSCs strongly expressed OV6, an antigen specific for rodent hepatic stem cells [19,20]. Taken together, these results suggest that the cells generated from SSCs expressed both hepatocyte and cholangiocyte markers, indicating that they retained the bipotential nature of hepatic stem cells. Notably, we found the cells derived from SSCs didn’t express the ES cell markers, including SSEA-1, SSEA-4, Nanog, or TRA-1-81, suggesting that these cells are not reprogrammed to ES cells.

Significantly, Nodal, Wnt3a, and bFGF induce a very high efficiency of SSC transdifferentiation into hepatic stem-like cells, as evidenced by our observations that more than 97% of cells derived from SSCs were positive for CK18. These growth factors play important roles during embryogenesis and organogenesis of the liver. Nodal plays an essential role at the earliest stages of endoderm formation, and Nodal regulates early endoderm development *in vivo*[25]. The Wnt pathway is required for liver growth and development [26]. Wnt3a promotes endodermal induction from human iPS cells [9], and it is important for hepatic cell function. Wnt3a is present at critical stages of human liver development and it elicits a rapid and efficient cellular progression of iPS cells to hepatic endoderm [27]. bFGF is secreted by the mesoderm and it is the first factor that commits the foregut endoderm to form liver primordium [28]. Mesodermal cytokines induce foregut specification into hepatic endoderm and followed by FGF signaling into the liver bud [29,30]. This is the first report showing that Nodal synergized with Wnt3a and bFGF to induce SSC transdifferentiation into hepatic stem-like cells.

HGF is required for liver development and it is associated with the ontogenesis of liver [31]. Hepatocyte differentiation can be induced from mesenchymal stem cells by HGF and other growth factors [32]. Consistent with these findings, here we found that HGF and HCM could efficiently induce hepatic stem-like cells derived from SSCs to differentiate into small hepatocytes. Morphologically and phenotypically different from hepatic stem cells, small hepatocytes were round and had round nuclei but did not express CK19. Meanwhile, small hepatocytes expressed *Ck8*, *Ck18*, *Cyp7a1*, *Hnf3b*, and *Alb* transcript, suggesting that they possess partial characteristics of hepatocytes. HGF promotes hepatic growth and differentiation while OSM and Dex have been implicated in the maturation of the hepatocytes [32]. Here we found that HGF acted synergistically with OSM and Dex to effectively induce hepatic stem cells derived from SSCs to differentiate into cells with morphologic, phenotypic, and functional features of mature hepatocyte-like cells. In morphology, these cells became polygonal with a low nucleus/cytoplasm ratio. In phenotypes, these cells derived from C18-4 cells and primary SSCs expressed numerous hepatocyte markers, including CK8, *Ck18*, *Cyp7a1*, *Hnf4a*, *Hnf3b*, ALB, CYP1A2, *Tat*, and *Ttr*, but were negative for CK19 or *Ck7*, hallmarks for bile epithelial cells. Of great interest are our findings that the cells generated from SSCs had the functional attributes of mature hepatocyte-like cells since they could synthesize albumin, produce urea, as well as uptake and release ICG.

It is unclear what signaling pathways are involved in cellular transdifferentiation. ERK1/2 and Smad2 pathways regulate a variety of cellular functions, including cell proliferation, differentiation, and cell cycle progression [16,17]. We found that ERK1/2 and Smad2 but not Akt signaling pathways were activated during the transdifferentiation of SSCs into mature hepatocyte-like cells, which offers a novel insight into the mechanisms underlying liver development and stem cell reprogramming. The ability to transdifferentiate SSCs using patients’ own adult testis tissues directly into hepatic stem cells without having to go through de-differentiation to pluripotent ES-like cells and embryonic body formation is essential for reducing differentiation procedures and improving safety for their clinical applications. Our study not only outlines novel approaches for transdifferentiation of hepatic stem-like cells with high efficiency but also offers new methods for rapidly and directly differentiating hepatic stem cells into mature and functional hepatocyte-like cells, using only growth factors without genetic manipulation. As such, the approaches presented here could contribute to overall objective of using patient-specific SSCs to generate mature and functional hepatocytes for cell transplantation and tissue engineering for the treatment of liver-related diseases as well as for hepatotoxicity screening of pharmaceutical drug development.

## Conclusion

**Figure 9:**
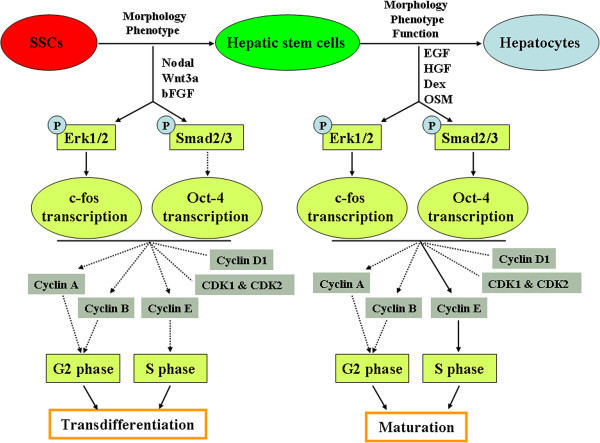
**Schematic diagram demonstrated the transdifferentiation of SSCs into mature hepatocyte-like cells and ERK1/2 and Smad2/3 signaling pathways.** “P” indicated “phosphorylate”; solid arrow denoted “promote”; dotted arrow indicated “inhibit”; dotted line showed “no change”.

In summary, we have for the first time demonstrated that SSCs have a greater plasticity to directly transdifferentiate into hepatic stem-like cells capable of differentiating into the cells with morphology, phenotypes and function of mature hepatocyte-like cells through the ERK1/2 and Smad2/3 signaling pathways and the inhibition of cyclin A, cyclin B and cyclin E (Figure 9). This study thus sheds novel insights into molecular mechanisms of stem cell reprogramming and liver development. Significantly, mature hepatocytes generated from a patient’s own SSCs could avoid immunological rejection. This study provides a novel strategy to generate mature and functional hepatocytes for cell therapies for liver diseases and drug toxicology screening.

## Competing interests

The authors declare no conflict of interest with the submitted paper.

## Authors’ contributions

ZH: conception and design, data analysis and interpretation, manuscript writing, financial support, final approval of manuscript; ZZ: carried out experiments, collection and assembly of data, manuscript writing, data analysis and interpretation; YG, YH, YG: participated in some experiments, Collection and assembly of data; YL: provision of study material; HY, SY, MM, LL: Collection and assembly of data; ZL, WQG: data analysis and interpretation, manuscript writing. All authors read and approved the final manuscript.

## Supplementary Material

Additional file 1: Table S1 Primers sequences used for RT-PCR. Click here for file

Additional file 2: Figure S1 Phenotypic characterization of the C18-4 cells. Immunocytochemistry showed expression of VASA (A), UCHL1 (B), GFRA1 (C), and RET (D) in the C18-4 cells. Scale bars in A, B, C, and D = 50 μm. Click here for file

Additional file 3: Figure S2 Morphological features of the cells derived from SSCs when cultured with various conditioned medium. Phase-contrast microscopy revealed the morphology of the cells from SSCs cultured with 10% FBS (A), 50 ng/ml Activin A + 50 ng/ml Wnt3a (B), 50 ng/ml Nodal + 50 ng/ml Wnt3a (C), 50 ng/ml Nodal + 50 ng/ml Wnt3a + 20 ng/ml bFGF + liver extract (D), 50 ng/ml Activin A + 50 ng/ml Wnt3a + liver extract (E), and 50 ng/ml Nodal + 50 ng/ml Wnt3a + liver extract (F). Scale bars in A-F = 50 μm. Click here for file

Additional file 4: Figure S3 Phenotypic characterization of the cells derived from C18-4 cells. Immunocytochemistry showed expression of VASA (A), RET (B), GFRA1 (C), and PLZF (D) in the cells generated from C18-4 cells. Scale bars in A, B, C, and D = 50 μm. Click here for file

Additional file 5: Figure S4 Phenotypic characterization of the cells derived from C18-4 cells. Immunocytochemistry showed expression of SSEA-1 (A), SSEA-4 (B), Nanog (C), and TRA-1-81 (D) in the cells generated from C18-4 cells. Scale bars in A, B, C, and D = 50 μm. Click here for file

Additional file 6: Figure S5 *Ck19* transcript and CK8 protein expression in SSCs, hepatic stem-like cells, small hepatocytes derived from SSCs. (A) RT-PCR revealed mRNA expression of *Ck19* in SSCs (lane 1), SSC induction for 7 days (lane 2), SSC induction for 10 days (lane 3), and small hepatocytes (lane 4). (B) Western blots showed CK8 expression in mature hepatocyte-like cells derived from SSCs (lane 1), SSCs (lane 2), and small hepatocytes derived from SSCs (lane 3). ACTB served as a loading control of total proteins. Click here for file
