# Supplementary material for: Direct transdifferentiation of spermatogonial stem cells to morphological, phenotypic and functional hepatocyte-like cells via the ERK1/2 and Smad2/3 signaling pathways and the inactivation of cyclin A, cyclin B and cyclin E
Source: Cell Commun Signal. 2013 Sep 18;11:67. doi: 10.1186/1478-811X-11-67 (PMC3848919; doi:10.1186/1478-811X-11-67)
Supplement: Additional file 1: Table S1 — Primers sequences used for RT-PCR. [file 1478-811X-11-67-S1.doc]

**Supplementary Table 1: Primers sequences used for RT-PCR**

| Gene | Primer sequence | Product size (bp) | Tm (°C) |
| --- | --- | --- | --- |
| *Alb* | Forward 5'-CCAAATTGGCAACAGACC-3'  Reverse 5'-TGCTCCACCTCACTAAGAC-3' | 190 | 55 |
| *CDK1* | Forward 5'-GCTCGTTACTCCACTCCG-3'  Reverse 5'-CCCTACGACCAGCAGACA-3' | 397 | 52 |
| *CDK2* | Forward 5'-ACAAGTTGACGGGAGAAGT-3'  Reverse 5'-TGAAGGACACGGTGAGAAT-3' | 307 | 52 |
| *c-fos* | Forward 5'-ATGATGTTCTCGGGTTTC-3'  Reverse 5'-CTTATTCCTTTCCCTTCG-3' | 444 | 56 |
| *Ck7* | Forward 5'-CAGCGTGCCAAGTTAGAGT-3'  Reverse 5'-GAGATATTCACAGGTCCCATT-3' | 260 | 51 |
| *Ck8* | Forward 5'-CGTCTGTGGTGCTGTCTATG-3'  Reverse 5'-TTGCGGTAGGTGGTGATC-3' | 454 | 51 |
| *Ck18* | Forward 5'-GTTGTCACCACCAAGTCTGC-3'  Reverse 5'-CCATCCACGATCTTACGG-3' | 407 | 51 |
| *Ck19* | Forward 5'-TCAGTACGCATTGGGTCA-3'  Reverse 5'-AGTAGGAGGCGAGACGAT-3' | 229 | 51 |
| *Cyclin A* | Forward 5'-CTGAGAATGGAGCACCTA-3'  Reverse 5'-TCTGTTGTGCCAATGACT-3' | 265 | 50 |
| *Cyclin B* | Forward 5'-GAGATGTACCCTCCAGAA-3'  Reverse 5'-CCATGTCGTAGTCCAGCA-3' | 232 | 50 |
| *Cyclin D1* | Forward 5'-CACAACGCACTTTCTTTCCA-3'  Reverse 5'-GACCAGCCTCTTCCTCCAC-3' | 164 | 55 |
| *Cyclin E* | Forward 5'-GATCGTTACATGGCATCAC-3'  Reverse 5'-CAATGGTCAGAGGGCTTA-3' | 220 | 50 |
| *Cyp1a2* | Forward 5'-CAGAGCGGTTTCTTACCA-3'  Reverse 5'-CCATAGTTGGGTGTCAGGT-3' | 202 | 55 |
| *Cyp7a1* | Forward 5'-CAATAGCCTGTCAACCAA-3'  Reverse 5'-ATCACCTCCAGCCTCTAC-3' | 319 | 55 |
| *Gapdh* | Forward 5'-TCTCCTGCGACTTCAACA-3'  Reverse 5'-TGGTCCAGGGTTTCTTACT-3' | 178 | 55 |
| *Hnf3b* | Forward 5'-AACTCCATCCGCCACTCT-3'  Reverse 5'-GTCTTCTTGCCTCCGCTA-3' | 218 | 55 |
| *Hnf4a* | Forward 5'-CTGCCAATATCGCTACAAC-3'  Reverse 5'-CATCCCTACGCTCCAGTA-3' | 449 | 55 |
| *Tat* | Forward 5'-CTGCCATTCCTGTACTCC-3'  Reverse 5'-CTCCAGCATCATCACCTC-3' | 215 | 51 |
| *Ttr* | Forward 5'-CCATCGCCACTACACCAT-3'  Reverse 5'-TCCTGAGCTGCTAACACG-3' | 182 | 51 |
