# Supplementary figures and images for: Direct transdifferentiation of spermatogonial stem cells to morphological, phenotypic and functional hepatocyte-like cells via the ERK1/2 and Smad2/3 signaling pathways and the inactivation of cyclin A, cyclin B and cyclin E
Source: Cell Commun Signal. 2013 Sep 18;11:67. doi: 10.1186/1478-811X-11-67 (PMC3848919; doi:10.1186/1478-811X-11-67)

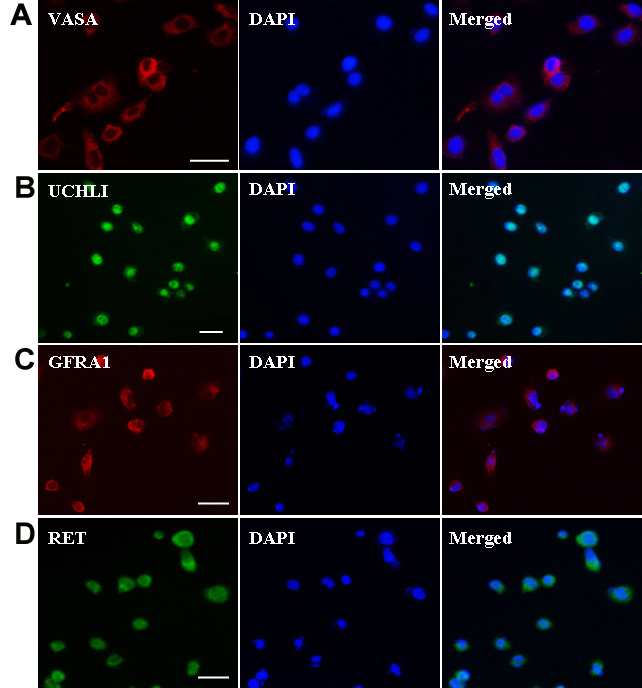

Supplement: Additional file 2: Figure S1 — Phenotypic characterization of the C18-4 cells. Immunocytochemistry showed expression of VASA (A), UCHL1 (B), GFRA1 (C), and RET (D) in the C18-4 cells. Scale bars in A, B, C, and D = 50 μm. [file 1478-811X-11-67-S2.jpeg]

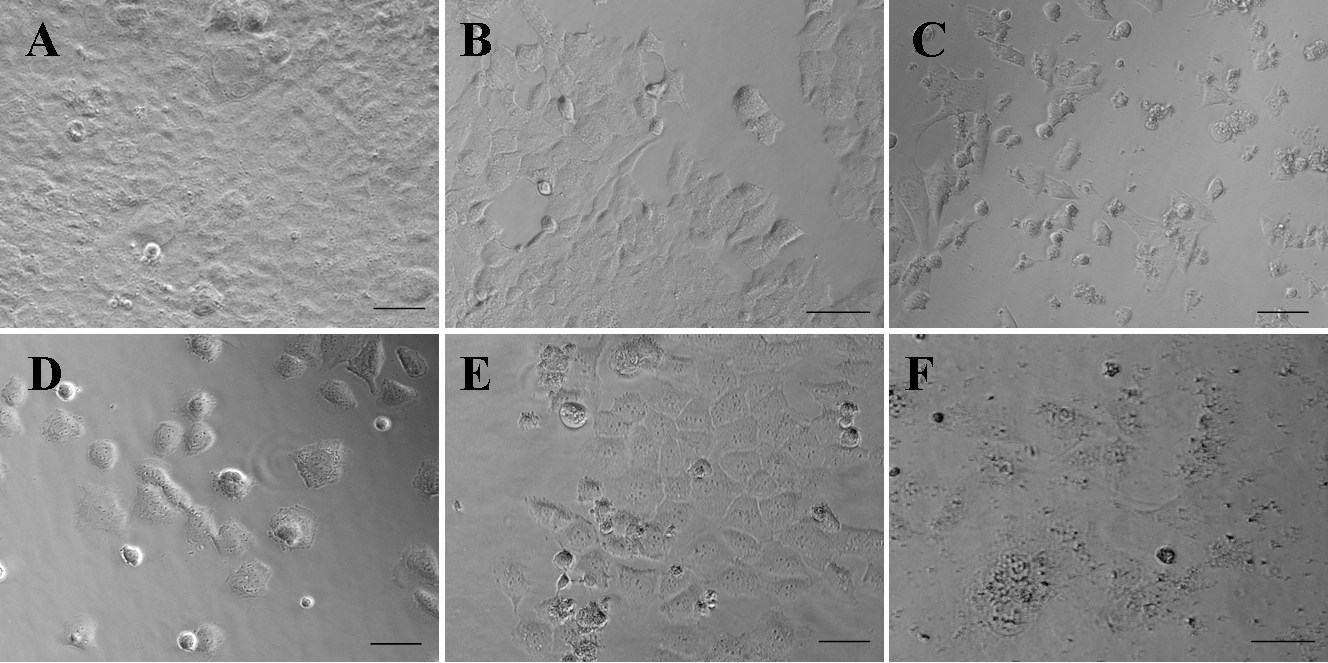

Supplement: Additional file 3: Figure S2 — Morphological features of the cells derived from SSCs when cultured with various conditioned medium. Phase-contrast microscopy revealed the morphology of the cells from SSCs cultured with 10% FBS (A), 50 ng/ml Activin A + 50 ng/ml Wnt3a (B), 50 ng/ml Nodal + 50 ng/ml Wnt3a (C), 50 ng/ml Nodal + 50 ng/ml Wnt3a + 20 ng/ml bFGF + liver extract (D), 50 ng/ml Activin A + 50 ng/ml Wnt3a + liver extract (E), and 50 ng/ml Nodal + 50 ng/ml Wnt3a + liver extract (F). Scale bars in A-F = 50 μm. [file 1478-811X-11-67-S3.jpeg]

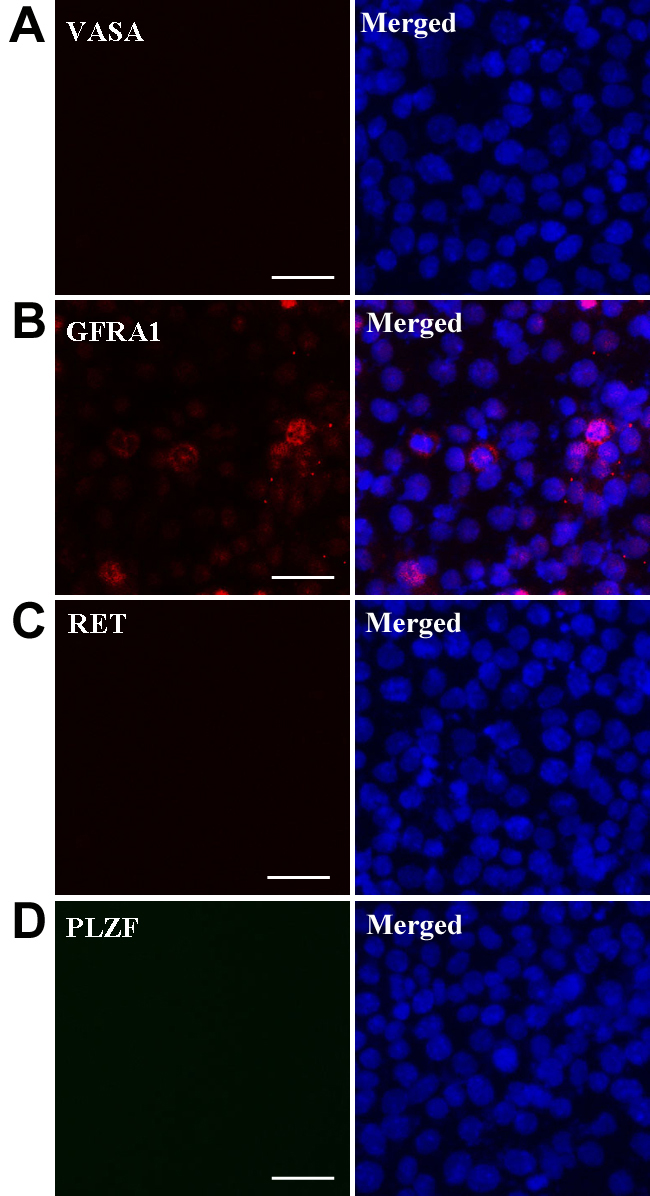

Supplement: Additional file 4: Figure S3 — Phenotypic characterization of the cells derived from C18-4 cells. Immunocytochemistry showed expression of VASA (A), RET (B), GFRA1 (C), and PLZF (D) in the cells generated from C18-4 cells. Scale bars in A, B, C, and D = 50 μm. [file 1478-811X-11-67-S4.jpeg]

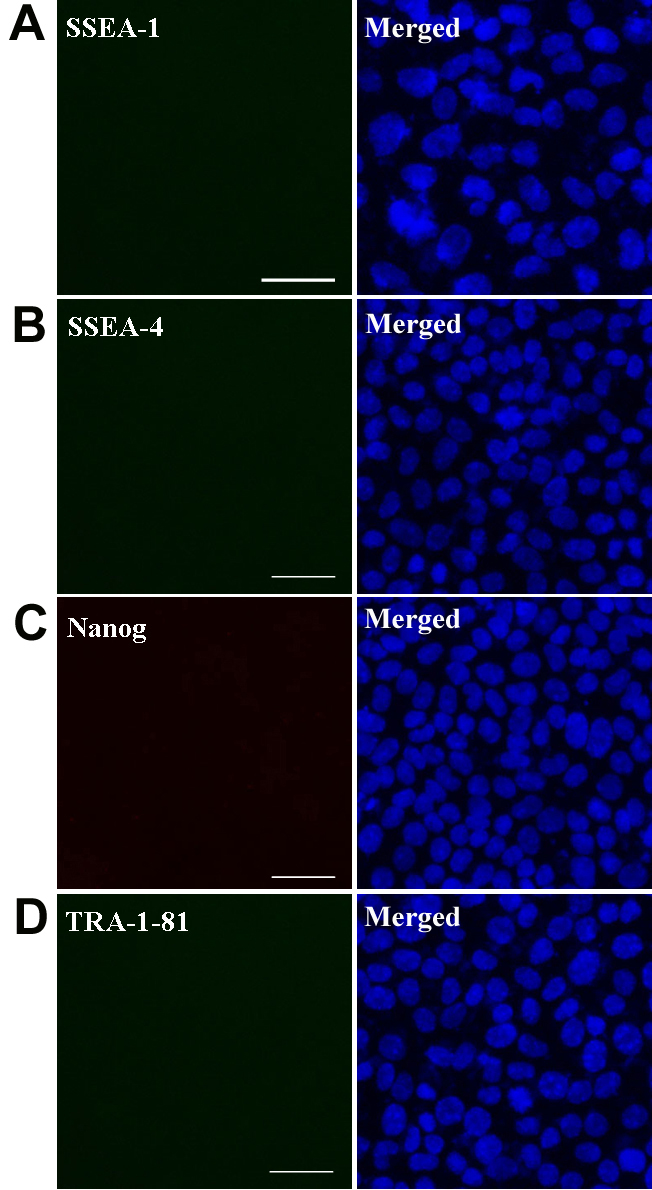

Supplement: Additional file 5: Figure S4 — Phenotypic characterization of the cells derived from C18-4 cells. Immunocytochemistry showed expression of SSEA-1 (A), SSEA-4 (B), Nanog (C), and TRA-1-81 (D) in the cells generated from C18-4 cells. Scale bars in A, B, C, and D = 50 μm. [file 1478-811X-11-67-S5.jpeg]

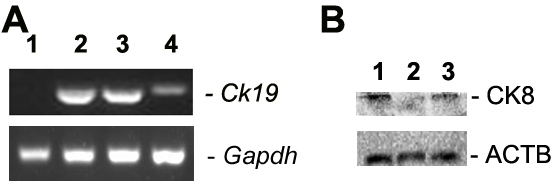

Supplement: Additional file 6: Figure S5 — Ck19 transcript and CK8 protein expression in SSCs, hepatic stem-like cells, small hepatocytes derived from SSCs. (A) RT-PCR revealed mRNA expression of Ck19 in SSCs (lane 1), SSC induction for 7 days (lane 2), SSC induction for 10 days (lane 3), and small hepatocytes (lane 4). (B) Western blots showed CK8 expression in mature hepatocyte-like cells derived from SSCs (lane 1), SSCs (lane 2), and small hepatocytes derived from SSCs (lane 3). ACTB served as a loading control of total proteins. [file 1478-811X-11-67-S6.jpeg]
